# Isometric Arm Forces Exerted by Females at Different Levels of Physical Comfort and Their EEG Signatures †

**DOI:** 10.3390/brainsci13071027

**Published:** 2023-07-04

**Authors:** Mahjabeen Rahman, Waldemar Karwowski, Nabin Sapkota, Lina Ismail, Ashraf Alhujailli, Raul Fernandez Sumano, P. A. Hancock

**Affiliations:** 1 Computational Neuroergonomics Laboratory, Department of Industrial Engineering and Management Systems, University of Central Florida, Orlando, FL 32816, USA; wkar@ucf.edu; 2 Department of Engineering Technology, Northwestern State University of Louisiana, Natchitoches, LA 71497, USA; sapkotan@nsula.edu; 3 Department of Industrial and Management Engineering, Arab Academy for Science, Technology, and Maritime Transport, Alexandria 2913, Egypt; linaelsherif@knights.ucf.edu; 4 Department of Management Science, Yanbu Industrial College, Yanbu 46452, Saudi Arabia; alhujaillia@rcyci.edu.sa; 5 Industrial Engineering Technology, Dunwoody College of Technology, Minneapolis, MN 55403, USA; rfsumano@knights.ucf.edu; 6 Department of Psychology, University of Central Florida, Orlando, FL 32816, USA; peter.hancock@ucf.edu

**Keywords:** physical exertions, arm, physical comfort, work design, EEG, power spectral density, neural signatures

## Abstract

A variety of subjective measures have traditionally been used to assess the perception of physical exertion at work and related body responses. However, the current understanding of physical comfort experienced at work is very limited. The main objective of this study was first to investigate the magnitude of isometric arm forces exerted by females at different levels of physical comfort measured on a new comfort scale and, second, to assess their corresponding neural signatures expressed in terms of power spectral density (PSD). The study assessed PSDs of four major electroencephalography (EEG) frequency bands, focusing on the brain regions controlling motor and perceptual processing. The results showed statistically significant differences in exerted arm forces and the rate of perceived exertion at the various levels of comfort. Significant differences in power spectrum density at different physical comfort levels were found for the beta EEG band. Such knowledge can be useful in incorporating female users’ force requirements in the design of consumer products, including tablets, laptops, and other hand-held information technology devices, as well as various industrial processes and work systems.

## 1. Introduction

### 1.1. Comfort at Work

Recently, increasing attention in the field of human factors and ergonomics has been given to the theoretical models and practical meaning of comfort at work [1,2]. It has been observed that many ergonomic investigations have considered negative aspects of work, such as physical discomfort, illness, or musculoskeletal disorders [3]. Therefore, a novel perspective on positive constructs, such as comfort and productivity, was proposed. The perception of comfort can be used as an effective design parameter for developing productive and efficient work environments [3]. 

Human perception of comfort is a complex subjective construct determined by an intricate interplay among psychological, spiritual, physical, social, and environmental factors [4]. The importance of investigating the perception of occupational tasks with consideration of cognitive aspects has been shown [5]. Because the voluntary control of muscle movements and motor activities is a crucial function of the brain, many studies have focused on the cerebral cortex, which is involved in controlling muscle activation processes [6]. A useful method for assessing brain activity at work is electroencephalography (EEG) [7]. In this study, we focus on the neural signatures of physical comfort.

A few published studies focus on the neural signatures of comfort related to working environments. Most of these studies investigated EEG signatures of comfort influenced by some element of the environment, e.g., the effect of clothing, outside temperature, exposure to light, driving a vehicle, etc., while participants were at rest or performing simple mental activities (see Table 1). 

Furthermore, studies on EEG signatures when participants engage in a physically demanding task are rather limited. Because the current research focuses on the EEG-based brain signatures of physical activity, relevant EEG-based investigations on isometric exertions with the upper limbs, such as hands, fingers, wrists, arms, and elbows, have also been reviewed. A summary of the key findings of these studies is presented in Table 2. 

### 1.2. Perception of Exertion and Comfort during Isometric Tasks

The perception of physical effort is subject to the psychophysical power law (Stevens, 1957) [25], which defines the relationship between the perceived intensity and the stimulus strength [5]. Accordingly, many studies have assessed the rate of physical exertion, on the basis of Borg’s scales [26]. For example, several studies have reported that movement related cortical potential (MRCP) correlates with force levels, force rate, fatigue, and muscle movement [27]. In another review, the role of emotion and fatigue along with other factors play a role in motor planning and execution [28]. In non-fatigued participants asked to perceive their level of physical effort, greater activity was observed in the prefrontal areas in addition to the motor cortex [29]. Similar results were reported by [30], in which the MRCP amplitudes correlated with perceived physical exertion, demonstrating central and peripheral muscle fatigue.

Other major findings included greater perceived exertion and physiological stress after the assigned leg exercise. After a leg-resistance exercise with cognitive tasks, a decrease in EEG oscillations and beta cortico-muscular coherence was observed [31]. Furthermore, an increase in theta power and a more active frontal region was reported by combining both physical and cognitive tasks; the perception of exertion significantly increased with the intensity of physical exercise [32]. Some of these investigations can be interpreted as indicating that a high level of physical comfort resulting from relaxing, low-intensity exercise is associated with an increase in alpha and theta spectral power [33,34]. However, a direct assessment of EEG signatures associated with isometric forces at different levels of physical comfort and their perception remains an unexplored area. 

### 1.3. Rationale for a Female-Centric Study

The sex of participants is an important factor that should be considered in studies at all levels of neuroscience. Several factors influence the differences between human male and female brains, including aging, functionality, hormone response, and wiring [35]. Based on the analyses of over 6000 participants’ EEG data collected during two different tasks in an uncontrolled environment, robust sex differences in several EEG measures were reported [36]. They also discussed how the age-related shifts in EEG activity differ between males and females. Notably, a deep learning algorithm demonstrated sex-specific differences in human brain signals [37]. Recently, Cave et al. (2021) reported significant differences in neuronal activity measured by resting EEG between young adult females and males and concluded that future EEG research should consider sex as a potential confounding variable [38]. On the physical side of human performance, significant gender differences were found to exist in EEG recorded during hand movement [39]. Specifically, they reported greater power decreases for females in all analyzed EEG frequency bands in topographic maps located in the centro-parietal brain area compared to males.

Beyond the brain, significant differences exist in physical strength, perception, and judgment between males and females [40,41]. Furthermore, prior literature surveys have revealed a substantial data gap in investigations of EEG indices for physical activity and the overall published scientific research on the female population [42,43]. Therefore, this study focuses on female subjects only. We note that some EEG studies utilizing only female participants have also been reported in the past. For instance, EEG correlates of working memory performance were investigated in females only [44]. However, we would like to note that our subsequent study explores the same research questions in male participants.

### 1.4. Objectives

Presently, the understanding of physical comfort experienced at work is rather limited. The main objective of this study was first to investigate the levels of isometric arm forces exerted by females at different levels of physical comfort and second to assess their corresponding neural signatures expressed in EEG power spectral density (PSD). The study examined the PSDs of four major EEG frequency bands in the brain regions controlling motor and perceptual processing. We hypothesized that differences would exist in the forces exerted by females and the corresponding EEG indices of brain activity, owing to the different levels of voluntary isometric arm exertion exhibited by the participants at different predefined physical comfort levels, ranging from *very low* to *very high*. 

## 2. Methods and Procedures

### 2.1. Participants 

Eight healthy female adults (27.5 ± 7.7 years old; body weight: 58.1 ± 10.9 kg; and body height 162.6 ± 6.44 cm) participated in this study. It should be noted that other similar EEG studies have included fewer than eight participants [45,46]. The participation exclusion criteria were any history of cardiovascular disease; neural or psychological disorders; polycystic ovarian syndrome; back pain; or any other serious illnesses and injuries. None of the participants were pregnant at the time of the experiment. The experimental procedures were approved by the Institutional Review Board (IRB) at the University of Central Florida. Written informed consent from all participants was obtained before the experiment. The participants were familiarized with the experimental procedures before the trials began. 

### 2.2. Experimental Design

**Figure 1:**
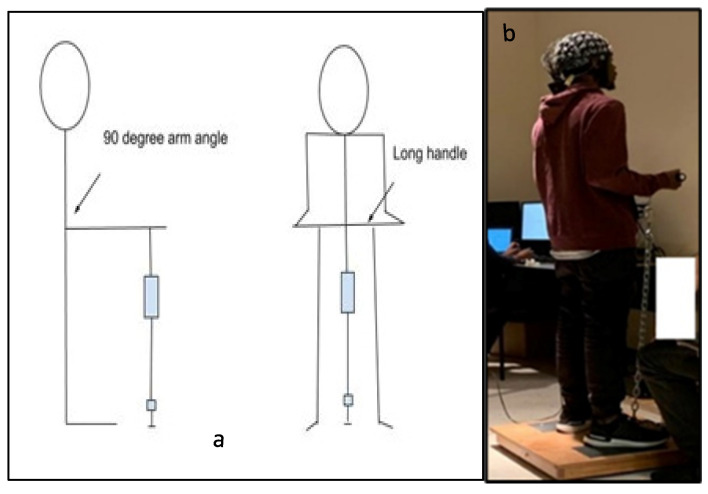
(**a**) Schematic diagram of the side and front views of an isometric arm exertion by using the Jackson strength evaluation system [47]. (**b**) Photograph from an experimental session. Figure 1**a**. Alt text: An illustration depicting the participants’ standing posture illustrating how the arm exertions were performed in the laboratory. Part (**a**) is a schematic diagram of an isometric arm exertion’s side and front views. Part (**b**) is a photograph showing the subject with the electroencephalography (EEG) cap on his head during the experimental session.

**Figure 2:**
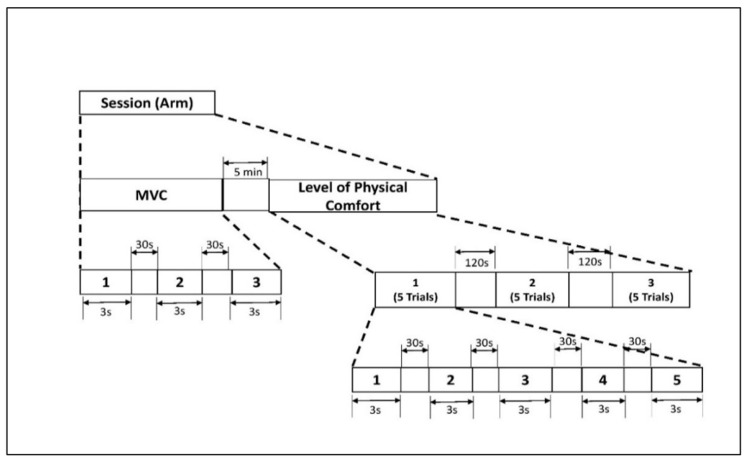
Experimental protocol for isometric arm exertion. Figure 2. Alt text: An illustration with a flow diagram depicting the experimental timeline of isometric arm exertion. There are two stages: maximum voluntary contraction (MVC) and level of physical comfort. MVC includes 3 trials of 3 s with 30 s rest. Level of physical comfort has 5 trials of 3 s with 30 s rest in between. This is repeated three times, and they are a 120 s rest period apart.

The experimental protocol included isometric arm exertions at different levels of physical comfort [47]. To assess the level of maximum voluntary contraction (MVC), we asked the participants to increase the exerted force to the maximum possible level without jerking and to maintain the exertion for 3 s [48]. An average of three MVC trials were recorded. Next, the participants performed isometric arm exertion in the same posture (see Figure 1) at five predefined levels of physical comfort: *very low* (VL), *fair* (F), *moderate* (M), *high* (HH), and *very high* (VH). In each experimental set, the comfort levels were applied in random order to minimize learning effects. Thirty seconds of rest was applied between all experimental sessions. The assigned levels of comfort were selected from the proposed 11-point unipolar rating of the perceived physical comfort scale (see Table A1 in the Appendix A). This scale was developed based on the assumption that the level of physical comfort is associated with a corresponding magnitude of physical exertion [49]. For each exertion, the exerted force was measured with a Torbal^TM^ FC 5k series force meter (Scientific Industries, Inc., Bohemia, NY, USA). After each exertion, the participants were also asked to assess their physical effort on Borg’s scale of RPE [26]. The detailed study protocol is shown in Figure 2.

### 2.3. EEG Data Acquisition

Brain activity during isometric exertion was recorded with a Cognionics Data Acquisition Software Suite and 64 channel Cognionics Mobile-64 device (CGX, San Diego, CA, USA). Conductive AgCl gel was used to create an electrical connection with the scalp. The device electrodes were arranged in a 10–10 configuration. The electrode contact impedance was carefully monitored and maintained at the lowest level possible, i.e., 20 k Ohm or less [50]. Before the experiment, the participants were trained in how to minimize artifacts such as eye blinking, tongue movement, and any other facial movements. They were also shown how these activities affect EEG recordings. Before recording, a cap in the most fitted size available was placed on each participant’s head, and gel was applied through syringes to each electrode. The impedance was then decreased for each electrode individually via a circular movement with a sterilized blunt needle tip. The entire process lasted approximately 30–40 min. After the desired impedance level was reached, EEG signals were recorded at a sampling rate of 500/s. 

### 2.4. EEG Data Preprocessing

EEG data processing was performed in the EEGLAB toolbox of MATLAB (The MathWorks, Inc., Natick, MA, USA) an open-source processing tool [51]. The acquired EEG data were resampled and band-pass filtered from 1 to 50 Hz. In the next step, clean_rawdata and artifact subspace reconstruction algorithms were applied to reject bad channels and correct continuous data, respectively [51,52]. The channels were also verified manually and removed if necessary, according to channel power spectra plots. The removed channels were interpolated, and average referencing was then applied. The continuous data were epoched between −1 and 4 s of the exertion performed. Epoch rejection tools, using an amplitude threshold from −500 to 500 µV, and improbability tests (6 SD for single channels, 2 SD for all channels) were applied. Adaptive mixture independent component analysis was used to separate independent components of the data that indicated better results than other ICA algorithms [53]. Finally, the IClabel plugin was applied to label and remove the artifactual components [54]. For all steps in data preprocessing, Makoto’s preprocessing guidelines and EEGLAB tutorials were used as guidelines [55].

### 2.5. EEG Data Analysis

**Figure 3:**
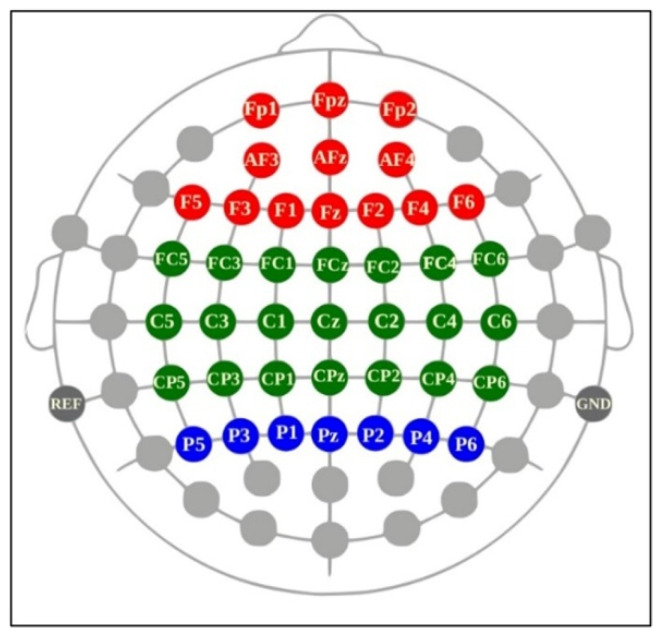
Categorization of selected channels from a Cognionics Mobile-64 headset: frontal (AFz, AF3–F4; Fz; F1–F6), central (FCz, FC1–FC6; Cz, C1–C6; CPz, CP1–CP6), and parietal (Pz; P1–P6) channels (adapted from Cognionics Inc.). Figure 3. Alt text: This brain map illustrates the location of all EEG electrodes from a Cognionics Mobile-64 headset according to the brain regions. Red, green, and blue correspond to frontal (AFz, AF3–F4; Fz; F1–F6), central (FCz, FC1–FC6; Cz, C1–C6; CPz, CP1–CP6), and parietal (Pz; P1–P6) channels, respectively.

Because cognition, motor planning, attention, and perception are executed in the frontal, central motor, and parietal cortices [56], we selected 41 channels covering these regions for potential analysis. The analyzed EEG band frequencies were theta (4–8 Hz), alpha (8–13 Hz), beta (13–30 Hz), and gamma (30–50 Hz) [57]. Alpha waves are inversely associated with cortical activation and represent a relaxed state [58]. Theta, beta, and gamma bands are primarily associated with motivational processes, memory, and emotion; sensorimotor behavior; and attention and object representation, respectively [59]. Although EEG has a high temporal resolution, the selected channels have typically been used in prior research for the investigation of the brain motor cortex and cognitive functions [60]. The region-based classification of the channels used in this study is illustrated in Figure 3. The PSD for all major brain wave frequencies was computed with MATLAB. PSD is an estimate of total power distribution over the frequency [61]. Thus, the PSD, or power spectra, measures the power of a signal for a certain frequency domain.

### 2.6. Statistical Analysis

Analysis of variance (ANOVA) and boxplots assessed the differences in RPE scores and exerted arm forces for different comfort levels. The correlation between arm forces and EEG PSD values at different levels of comfort has also been investigated. These analyses were performed for theta, alpha, beta, and gamma power. Since the observed EEG signatures were not normally distributed, non-parametric tests were applied to assess the changes in brain activation related to arm isometric exertions, both the relative and normalized PSD of all four EEG frequency bands.

## 3. Results

### 3.1. MVC and Forces Exerted at Different Levels of Physical Comfort 

**Figure 4:**
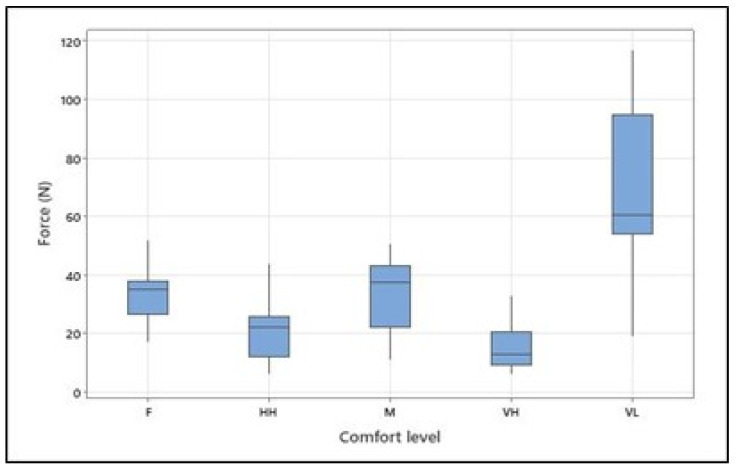
Isometrically generated arm forces at different levels of physical comfort (VL, F, M, HH, and VH correspond to *very low*, *fair*, *moderate, high*, and *very high*, respectively). Figure 4. Alt text: Force at different levels of physical comfort across all participants.

The overall mean, standard deviation, and range (min–max) of the maximum voluntary contraction [N] for arm flexion for all participants were 119.5, 52.86, and 37-204 [N], respectively. The means (and standard deviations) of the exerted forces [N] were as follows: 68.07 (28.58) for *very low* comfort, 32.77 (9.66) for *fair* comfort, 34.7 (11.04) for *moderate* comfort, 20.83 (7.97) for *high* comfort, and 14.99 (7.02) for the *very high* level of physical comfort (see Table 3 and Figure 4). As hypothesized, the ANOVA (see Table 4) revealed that the isometric arm forces exerted at different levels of physical comfort significantly differed (F = 19.57; *p* < 0.001). The effect size of 0.177 for “subject” indicates that individual differences among subjects explain only about 17.7% of the variation in the exerted forces. On the other hand, the effect size of 0.606 for “Comfort Level” suggests that the different comfort levels explain approximately 60.6% of the variation in force, making comfort level a strong predictor of force in this analysis. Tukey’s pairwise comparison of the means with a 95% confidence level confirmed the significant differences between forces exerted at *very low* and all other comfort levels, between *fair* and *very high*, and between *moderate* and *very high* comfort levels. 

### 3.2. Rate of Perceived Exertion at Different Levels of Physical Comfort

**Figure 5:**
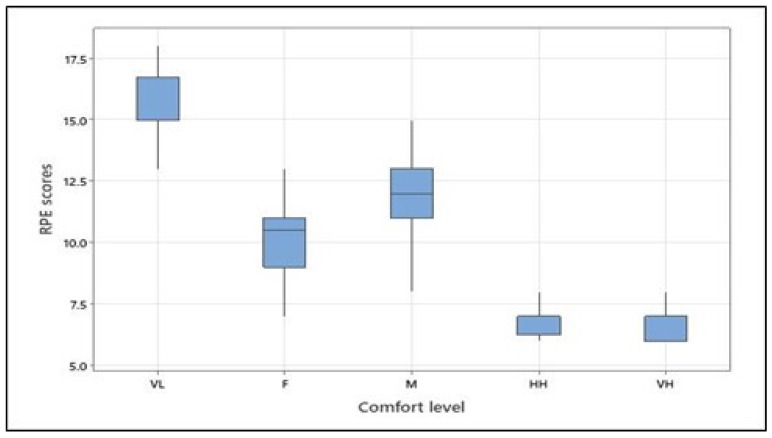
RPE scores at different levels of physical comfort across all participants (VL, F, M, HH, and VH correspond to *very low*, *fair*, *moderate, high*, and *very high*, respectively). Figure 5. Alt text: Rate of perceived exertion scores at different levels of physical comfort across all participants.

The ANOVA revealed that the rate of perceived exertion (RPE) at different levels of predefined physical comfort significantly differed (see Table 5). The means (and standard deviations) of the RPE scores were as follows: 15.46 (1.79) for very low comfort, 10.00 (1.77) for fair comfort, 12.08 (1.53) for moderate comfort, 7.04 (0.86) for high comfort, and 6.46 (0.59) for the very high level of physical comfort. Tukey’s pairwise comparison confirmed differences between RPE scores for all comfort levels with a 95% confidence level, except for *high* and *very high* levels, which did not statistically differ. It should be noted that for both the arm forces and the RPE scores, the *moderate* comfort category was higher than the *fair* comfort category, thus indicating that the participants perceived and applied more effort for the *moderate* than the *fair* physical comfort level (see Figure 5).

## 4. Neural Signatures of Force Exertion 

### 4.1. Brain Topographic Map

**Figure 6:**
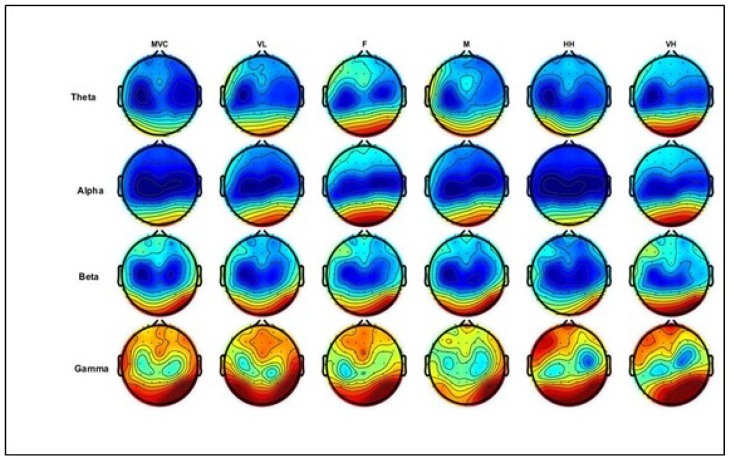
Illustration of grand average topographic head maps of EEG spectral power (dB) for theta, alpha, beta, and gamma for the MVC (average of three trials) and different physical comfort levels. Blue and red colors indicate the minima and maxima of the corresponding scales, respectively. The columns represent levels of comfort and MVC, whereas the rows denote EEG power from different frequency bands. Comfort levels: VL, F, M, HH, and VH correspond to *very low*, *fair*, *moderate*, *high*, and *very high*, respectively (shown in columns from left to right; MVC is in the first column). Figure 6. Alt text: This figure illustrates topographic head maps of EEG spectral power (dB) for theta, alpha, beta, and gamma for the MVC (average of three trials) and different physical comfort levels. Blue and red colors indicate the minima and maxima of the corresponding scales, respectively.

Figure 6 below illustrates the topographic head mapping, representing EEG power spectrum density distributions at the maximum voluntary contraction level (MVC) and different levels of physical comfort. The columns represent MVC and physical comfort levels, whereas the rows denote the EEG power at different frequency bands. In these examples, theta and alpha power differ in the frontal and parietal areas, while beta and gamma power show differences in the frontal area, parts of the central area, and the parietal brain areas. 

### 4.2. PSD–Force Correlation Analysis

Pearson correlation coefficients of the EEG PSD values and arm forces at different levels of physical comfort across all channels and all participants were calculated. The alpha power significantly (*p* < 0.05) correlated with force at a high comfort level. Beta power and force were significantly correlated at very low and moderate comfort levels. The theta power significantly (*p* < 0.05) correlated with force when the comfort levels were moderate and high. Finally, gamma power was significantly (*p* < 0.05) correlated with force at very low, moderate, and high comfort levels. Furthermore, the following significant correlations (*p* < 0.05) between the PSD values and arm forces at different levels of physical comfort were found. At the very low physical comfort level, both beta power (FC4: r = 0. 871) and gamma power (FC4: r = 0.921) correlated with the exerted forces in the central brain region. At the moderate level of physical comfort, beta power (P5: r = 0.774) and theta power (P5: r = 0.707) were correlated with the exerted forces in the parietal region. In addition, gamma power was correlated with force (CP4: r = 0.752) in the central region, and theta power was correlated with force (AF3: r = 0.733) in the frontal brain region. At the high level of physical comfort, alpha power was correlated with force in the central (CP5: r = 0.712) and parietal brain regions (P3: r = 0.724 and P5: r = 0.792), while gamma power was correlated with force in the frontal region (F5: r = 0.727) as well as the parietal region (P1: r = 0.717 and P5: r = 0.821). Finally, theta power was correlated with force in the frontal (AF3: r = 0.721) and parietal regions (P3: r = 0.775; P5: r = 0.708; and P6: r = 0.704).

The above results are in general agreement with findings from other studies reporting that EEG signatures were correlated with force level [19,27]. At *very low* comfort, the exerted force correlated with the beta and gamma power in the central channel, thus reflecting motor activity [21,62]. In our study, at the *moderate* comfort level, a positive correlation coefficient was found with theta power in the frontal region, theta and beta power in the parietal region, and gamma power in the central channels. Therefore, *moderate* comfort resulted in more attentional, sensorimotor, and perceptual effort along with force [63,64]. Finally, when the comfort level was *high*, theta power significantly correlated with force in the frontal and parietal areas, alpha power in the central and parietal areas, and gamma power in the frontal and parietal channels. Furthermore, the parietal channels showed significant force–EEG power correlation at the *high* and *moderate* comfort levels. Because the parietal region is responsible for attention, perception, and awareness, these correlations appear valid [56].

### 4.3. Relative and Normalized Power Spectrum Density 

As proposed in the subject literature, relative PSD, defined as the PSD ratio of the specific frequency band of the analysis target with that of the total frequency band, was used [65]. Application of the relative PSD values allows for a reduction in the inter-subject variability associated with absolute EEG signal power. The relative PSD changes of the EEG band were used to reduce inter-subject variance [66]. The change in relative PSD (RPSD) for each EEG channel was calculated using equation (1):

RPSD*_fi_* = *PSD_fi_*/*PSD_Total f_*_(*A*+*B*+*G*+*T*)_ (1)

where *f_i_* indicates specific frequency band (*i*) (*A = alpha*, *B = beta*; *G = gamma*, and *T = theta*) and *Total_f_* indicates the sum of PSD values over all frequency bands. The total RPSD*_fi_* value for the whole brain was defined as the sum across all EEG channels. 

As discussed by [65], since the relative PSD reflects changes in only one frequency band, as opposed to a totality of the effect of all frequency bands, it can be less accurate to assess the overall changes in the brain. Therefore, for the purpose of this study, we have also introduced the normalized PSD index. This was also to control for differences in the arm strength of all participants, and the potential effect of MVC on neural brain responses at the maximum voluntary contraction MVC level. The normalized PSD index (NPSD) for each EEG channel (electrode) was defined as a ratio of specific *PSD_fi_* values divided by the PSD corresponding to the maximum voluntary contraction (MVC), denoted as *PSD_fi MAX_* for each subject using the following Equation (2):

NPSD_*fi*_ = *PSD*_*fi*_/*PSD*_*fi MAX*_ (2)

This MVC-normalized index (NPSD*_fi_*) was also used to control for differences in the arm strength of all participants and the potential effect on neural brain responses at the maximum voluntary contraction MVC level. It should be noted that the above index is analog to normalization of the exerted forces which are commonly expressed as the % of the corresponding MVC values [67].

#### 4.3.1. Relative PSD Analysis

**Figure 7:**
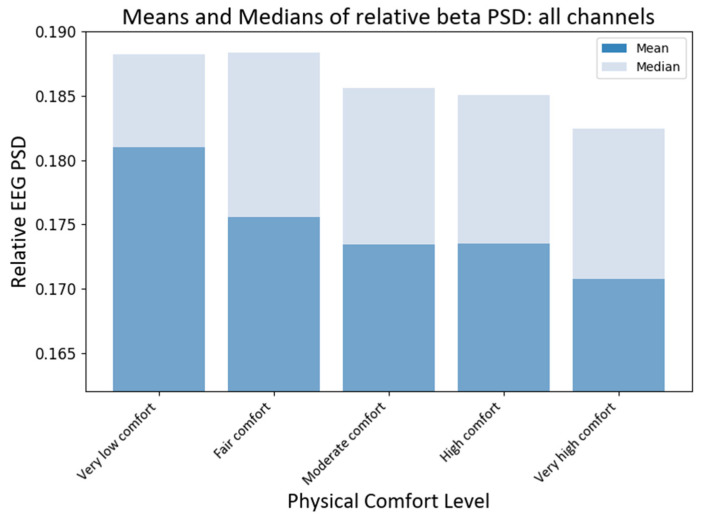
Grand average values of RPSD beta power at different levels of physical comfort. Figure 7. Alt text: This is a bar chart depicting the grand average values of the relative beta power with vertical bars at different levels of physical comfort.

To compare the effects of different levels of physical comfort on brain neural activity, we first compared the medians of the RPSD index of neural responses for all EEG channels (electrodes). The non-parametric Kruskal–Wallis (KW) test was chosen since the collected EEG data do not satisfy the normality assumption. Using the KW test, we could make valid statistical inferences without the need for normally distributed data. The KW test showed that the medians of different comfort groups significantly differed for beta PSD (H statistic = 15.58; *p*-value < 0.0037). The subsequent pairwise medians comparisons with grouping information were carried out using Dunn’s test [68] at a 95% confidence level. Python package scikit-posthocs version 0.7.0 was used to implement this test to compare the RPSD medians at different levels of physical comfort (see Figure 7). The median RPSD values for the beta PSD for *very* low–*moderate* and *very low–very high* levels of comfort were significantly different (*p*-values 0.017, and 0.0001, respectively) as per Dunn’s pairwise comparison test with a 95% confidence level.

#### 4.3.2. MVC-Normalized PSD Analysis

**Figure 8:**
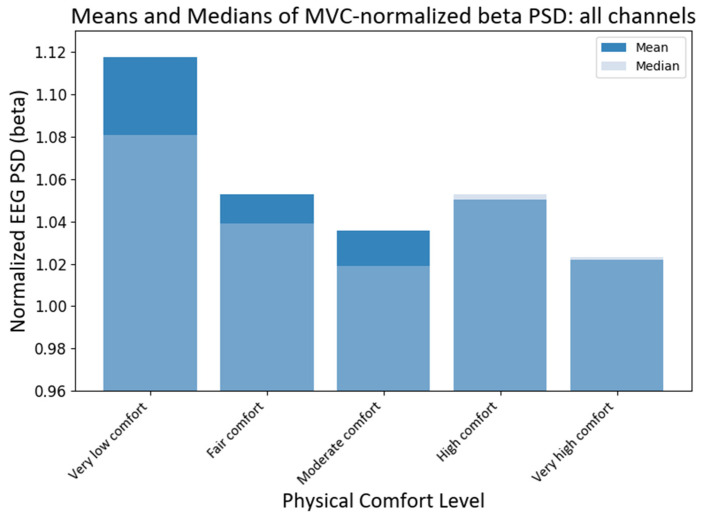
Grand average and median values of NPSD beta power at different levels of physical comfort. Figure 8. Alt text: This bar chart depicts the grand average and median values of the normalized beta power with vertical bars at different levels of physical comfort.

Next, we analyzed the MVC-normalized power (NPSD*_fi_* ) index of neural responses at different comfort levels across all EEG channels (electrodes). As before, the non-parametric Kruskal–Wallis (KW) was applied. The test showed that the medians of different comfort groups were significantly different for alpha PSD (H statistic = 18.63; *p*-value < 0.00093) and beta PSD (H statistic = 69.71; *p*-value ≈ 0). The subsequent pairwise medians comparisons with grouping information using Dunn’s test at a 95% confidence level were used to compare the NPSD medians at different levels of physical comfort (see Figure 8). The median NPSD value for the beta PSD was very low and was different from all the rest of the comfort levels (*p*-values < 0.00005) as per Dunn’s pairwise comparison test with a 95% confidence. Similarly, the median NPSD value for the alpha PSD for very low comfort differed from the high comfort level (*p*-value < 0.0098) as per Dunn’s pairwise comparison test with a 95% confidence level. 

**Figure 9:**
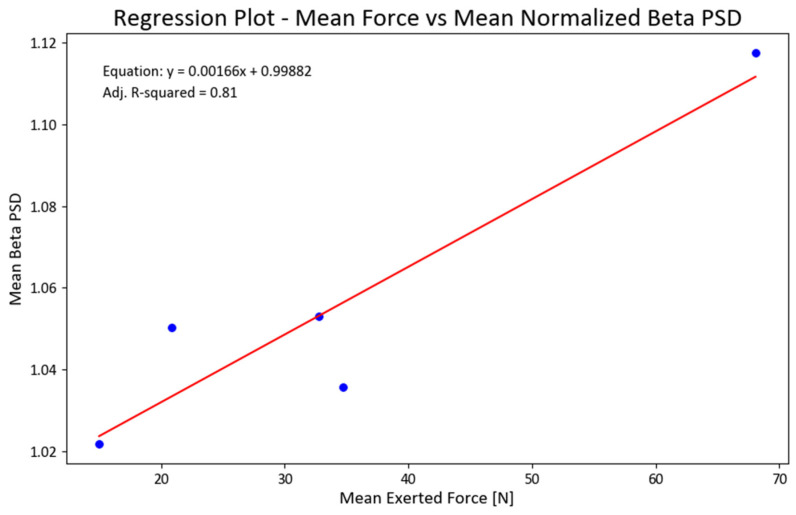
Regression between mean exerted forces (F) and mean NPSD values for beta PSD (NPSD_beta_ = 0.99882 + 0.00166(F); adjusted R^2^ = 0.81). Figure 9. Alt text: This chart illustrates a linear regression line for the mean exerted forces and normalized beta power.

We also ran a regression analysis between the mean forces and total NPSD for all EEG channels. The obtained linear regression demonstrated statistical significance (F = 18.61; *p* < 0.022) and accounted for 81% of the variance in the data (refer to Figure 9). To ensure the fulfillment of underlying regression assumptions, we conducted tests for the normality of residuals, heteroscedasticity, and multicollinearity. Considering the limited sample size, we examined the skewness and kurtosis of the beta PSD data used in the regression, resulting in values of −0.4 and 2.4, respectively. 

These findings alleviated our concerns about the adequacy and validity of the data, aligning with our commitment to ensure its reliability. Furthermore, the regression analysis for the gamma PSD exhibited a favorable fit (adjusted R-squared value of 63%), with skewness and kurtosis values of 0.76 and 2.5 for the PSD, respectively. However, the regression models for the other two bands did not yield statistically significant results.

### 4.4. MVC-Normalized PSD Analysis for the Motor Cortex

**Figure 10:**
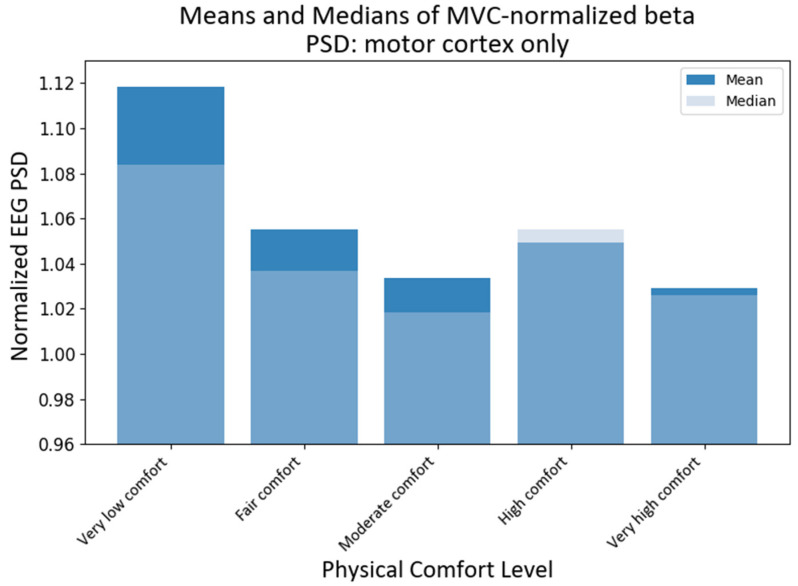
Grand average and median values of NPSD beta for the motor cortex only at different levels of physical comfort. Figure 10. Alt text: This bar chart depicts the grand average and median values of NPSD beta for the motor cortex only at different levels of physical comfort.

Since the motor cortex plays a significant role in controlling human exertion, even though EEG has a low spatial resolution, we also investigated the activity of those channels that have been associated with the motor cortex [69]. Assuming that the primary motor cortex lies anterior to the central sulcus that divides the frontal and parietal lobes [70], for the purpose of this study, we proposed that motor activity could be monitored predominantly by the following fifteen channels: C3, Cz, C4, C5, C6, CP3, CP5, CP6, Cz, FC3, FC4, FC5, FC6, FCz, and Pz according to the international modified 10–20 international system [71]. It should be noted that some of these channels have been used in the past to assess changes in upper extremity function and dynamic changes in EEG power [65,72]. The KW tests showed that the medians of different comfort groups were significantly different for alpha PSD (H statistic = 13.46; *p*-value < 0.0092) and beta PSD (H statistic = 46.96, *p*-value ≈ 0) when only the motor cortex region was chosen. The subsequent pairwise medians comparisons with grouping information using Dunn’s test at a 95% confidence level were used to compare the NPSD medians at different levels of physical comfort (see Figure 10). The median NPSD value for the beta PSD *for very low* was different from all of the rest of the comfort levels (*p*-values < 0.00029) as per Dunn’s pairwise comparison test with a 95% confidence level. However, upon conducting pairwise comparisons using Dunn’s method for alpha PSD, none of the comfort levels were found to be statistically significant at a 95% confidence level. 

## 5. Discussion

The main aim of this study was to assess the level of isometric arm forces exerted at predetermined levels of physical comfort. A proposed scale of physical comfort was applied for this purpose [49]. In addition, the neural signatures, as measured by EEG power spectrum density, at different comfort levels have been investigated. The results based on a limited number of participants suggest the presence of differences in exerted arm forces and RPE scores at the various levels of physical comfort. The isometric exertions applied in this study can be categorized as muscular activity with low mobility [43]. Previous research has also suggested that beta and gamma bands are higher during such physical activity, thus indicating motor circuit activation [21,62]. 

In general, frontal, central motor, and parietal beta rhythms have been associated with stimulus assessment, sensorimotor and movement control, and working memory accumulation, respectively [73,74]. The beta band frequency can also influence overall motor control [17,75]. While beta band activity has been investigated during simple and static muscular contractions [76], no previous studies have focused on neural correlates of physical comfort at the time of isometric exertion. 

Some of the prior studies reported significant relationships between EEG and the perception of effort, force levels, or exercise intensity [77,78]. Other studies found no significant changes in cortical activity with differing exercise intensity or force levels. The results of our study suggest no significant changes in alpha power at different levels of comfort in agreement with previous studies [24,79]. It should be pointed out that in our study, the participants were unaware of the level of comfort at which they would be asked to exert corresponding forces before any trial. This experimental design aimed to minimize the learning effect [80,81]. In addition, the current study’s short duration of isometric exertions might have caused a relatively low level of muscular fatigue [82]. The higher beta power observed in our study is consistent with the findings of other studies on physical exertion and physical activities requiring precision and decision-making [20]. The above also suggests that greater sensorimotor performance appears necessary to exert higher forces. Finally, it is also interesting to note that sex differences exist in every major part of the brain, and the sex of the participants is an important factor that should be considered in studies at all levels of neuroscience [83]. Furthermore, human scalp EEG recordings contain sex-specific information [37], and male brain rhythms may have more pronounced beta power than females. It is also plausible that females are more judicial when assessing load heaviness or postural discomfort at work than males [41,84].

Further investigations regarding neural signatures of the physical comfort of human behavior are needed to improve our understanding of the interrelationships between the biomechanical, physiological, psychological, and neural aspects of human physical performance at work [6,85]. Such knowledge can be very useful in workplace design that optimizes human physical and mental wellness at work. Future research should also include laboratory experiments involving many consecutive trials at the same physical comfort level to assess the effects of muscular fatigue. Comparisons among different age groups, fitness levels, sexes, and isometric tasks involving other body parts, e.g., the legs and torso, as well as isokinetic tasks, should be investigated. Finally, because the human brain is a very complex structure [86], nonlinear and functional connectivity analyses considering neural signatures of physical comfort and related force exertions should also be undertaken. While this small-scale research suggests that EEG power spectra indices can distinguish the isometric arm forces exerted at a “very low” level of physical comfort, it should be noted that the relationship between brain activity and force might be nonlinear [87,88]. Therefore, this study’s results are preliminary only and insufficient to fully assess the nature of neural signatures of human physical exertions at different levels of physical comfort at work.

## 6. Study Limitations

There are several important limitations of the study. First, this study employed a very small number of participants who were female only. Therefore, the presented results should be considered preliminary only and subject to confirmation in future research. We want to note that we have recently completed a follow-up study with a larger cohort of male participants. Second, we chose the sample size based on resource constraints regarding the limited number of available female participants for our laboratory experiments during the COVID-19 pandemic [89]. Third, although we minimized the potential learning effects in each participant by administering the different comfort levels in a random sequence unknown to the participants, the effects of potential learning effects have yet to be investigated. It should also be noted here that people may perform the same physical tasks repeatedly at work and in everyday life. Brain plasticity can affect neural signatures of body responses to such tasks [90,91]. Lastly, our laboratory study could have produced somewhat different results if a control condition with no force applied first was used. However, a substitute for the control provision was the application of the maximum voluntary contraction (MVC), which can be considered equivalent to a control condition. This is because, at first, each subject was asked to exert the maximum force to the best of her ability without stipulating any physical comfort level or other influence. Future studies should ruminate on the above considerations when investigating the neural signatures of physical exertions and the associated comfort of such exertions.

## 7. Conclusions

This study investigated changes in forces during isometric arm exertion and the perception of effort at different predefined physical comfort levels and their corresponding neural signatures expressed in EEG power spectrum density (PSD). To our knowledge, this is the first study considering physical comfort level as a factor in muscular activity. Given the significant study limitations, the current results indicated the presence of differences in exerted arm forces and RPE scores at the various physical comfort levels. The results also revealed higher beta activity at greater force exertions associated with the ‘very low’ level of physical comfort, indicating greater perceptual and sensorimotor efforts. Furthermore, we note that EEG signatures of isometric forces exerted at different levels of physical comfort can be affected by the compounded effects of psychological, sex-specific, and experimental factors. In addition to force, the perception of physical comfort at work can be influenced by many other factors [92,93]. The assessment of EEG correlates of force exertions should also consider the effects of perceptual and cognitive processes [94]. Moreover, we acknowledge that the reported EEG results could be partially affected by high individual differences among the participants and the limitations of power spectra density measures [95]. Future studies should include a much larger study of male and female populations to increase the power of the investigated relationships between the exerted forces, physical comfort, and their EEG signatures. 

## Figures and Tables

**Table 1:** Key findings of electroencephalography (EEG)-based studies of perceived comfort.

Comfort Type	Key Finding(s)
Visual comfort	EEG power related to the comfort of stereoscopic vision [8]. Level of illumination affected by EEG visual evoked potential [9].
Thermal comfort	Increase in theta and alpha power, decrease in beta power, and the presence of sensorimotor rhythm in a cool, comfortable indoor sitting environment [10]. Thermal comfort of sleep and its quality related to changes in EEG signals [11].
Wear comfort	Increased somatosensory activity in the brain’s frontal, parietal, and occipital regions with the pressure of a clothing girdle. Low alpha power at low comfort conditions [12].
Riding comfort	Correlation between EEG and subjective evaluation features in car riding comfort for different types of roads and tires [13].

**Table 2:** Key findings of EEG-based studies on isometric exertion with upper extremities.

Isometric Exertion Task	Key Finding(s)
Gripping or grasping	High levels of EEG activity during the preparation and onset of force exertion [14]. Decrease in the power of all EEG frequencies with muscle fatigue during the sustained exertion phase [15]. No effect of fatigue due to prolonged maximum voluntary contraction (MVC) on overall brain activation for controlling muscle movement [16]. Increase in theta and beta frequencies in different cortical regions during a gripping task with a dynamometer [17]. Increase in beta power at the start of the cue and a decrease in beta power during the preparation and task performance [18]. EEG signatures correlated with handgrip force levels [19].
Finger movement	Increased cortical activity in beta and gamma bands in the sensorimotor area with finger movement [20].
Wrist flexion and extension	Gamma band power allowed for the detection of motor circuit activation during rest or whilst performing tasks [21]. Lower peak beta cortico-muscular coherence and perceived difficulty for wrist flexion. Opposite results for wrist extension [22].
Elbow flexion	Significant decrease in EEG-EMG coherence in the beta band despite increased power [23].
Arm exertion	Lower alpha power for 1 kg and 3 kg loading in forearm exertions than under no load conditions. No significant differences between load levels. Significantly greater alpha power when tired than in the absence of fatigue [24].

**Table 3:** Isometric arm forces generated (and % MVC) at different levels of physical comfort.

Predefined Comfort Level	Arm Force (N)						
	Mean	SD	Range		% MVC		
			Min	Max	Mean	Minimum	Maximum
Very low comfort	68.08	28.58	19	117	56.97	15.89	97.9
Moderate comfort	34.70	11.04	11	51	29.04	9.21	42.68
Fair comfort	32.78	9.66	17	52	27.43	14.23	43.51
High comfort	20.84	9.79	6	44	17.44	5.02	36.82
Very high comfort	14.99	7.02	6	33	12.54	5.02	27.62

**Table 4:** ANOVA table of the effect of the comfort level on the exerted arm forces in newton [N].

Source	df	Adj SS	Adj MS	F-Value	*p*-Value
Subject	7	3964	566.3	3.26	0.0120
Comfort level	4	13,580	3394.9	19.57	<0.001
Error	28	4858	173.5		
Total	39	22,402			

**Table 5:** ANOVA for the effect of comfort level on the RPE scores.

Source	df	Adj SS	Adj MS	F-Value	*p*-Value
Subject	7	12.20	1.742	3.17	0.013
Comfort level	4	441.69	110.424	201.13	<0.001
Error	28	15.37	0.549		
Total	39	469.26			

## Data Availability

Data can be shared upon request from the corresponding author.

## Appendix A

**Table A1:** Rate of perceived physical comfort scale [49].

0	No Physical Comfort
1	Very low physical comfort
2	
3	Fair physical comfort
4	
5	Moderate physical comfort
6	More than moderate physical comfort
7	
8	High physical comfort
9	
10	Very high physical comfort
